# Silver Sutures in Surgery

**Published:** 1858-07

**Authors:** 


ART. IV.—*Silver Sutures in Surgery. The Anniversary Discourse before the New York Academy of Medicine.* By J. MARION SIMS, M.D., Surgeon to the Woman's Hospital. (Published by order of the Academy.) New York: S. S. & W. Wood, 1858.

THE name of the orator here presented, and the theme exhibited in his title-page, naturally arouse more practical attention than is usually accorded to an ephemeral address. The reader, however, who expects to find in this extravagant and absurdly egotistical discourse a scientific study of the advantages and use of pure silver as a material for sutures, will be sadly disappointed.

A mere special plea for personal aggrandizement, with a tirade against competitors and the most cursory allusion to the original labors of his predecessors, are not the demonstrations to be looked for in a contribution to the progress of operative surgery, formally invited, as the author intimates, by the members of the New York Academy of Medicine.

The manifesto of Dr. Sims, in answer to that call, is certainly a very different conclusion from what was confidently expected in the first fruits of the rare opportunities of the Surgeon to the Woman's Hospital, and which might have been offered to the Academy as an honorable tribute to the humanity and wisdom of the founders of that charity.

Were it not before us, in the black and white of a printed pamphlet, published "by order," etc., and with all its *ad captandum* paraphernalia of pictures and staring capitals, we should try to regard its various peculiarities as the harmless frothiness of an after-dinner effusion, easily accounted for and as readily forgotten. As it is, however, there is so much which demands explicit contradiction, that we find it impossible to confine our notice within the brief space to which such emanations are alone entitled.

We have no inclination for the discussion involved in the examination of its remarkable pages, nor have we any desire to increase the already industriously extended notoriety of what has, doubtless, ere this, proved a source of mortification, as it is of discredit, to all concerned in its publication. We should gladly pass it by, along with the periodical issues of a very different but not less enthusiastic order of seekers for popular *éclat,* and would have little but surprise and regret to express in relation to its misrepresentations and assumptions, were it not for the previous distinction of its author, and the authoritative auspices under which it is put forth.

Under these circumstances we are compelled, in defence of a grossly assaulted contributor to this journal, and for the credit of American Surgery, so far as our position as journalists involves us, to deny the shadow of a foundation for the charge of bad faith so piteously urged against Dr. Bozeman, and to repudiate the bombastic pretensions of Dr. Sims as to the importance and reality of his alleged "discovery" of the silver suture.

We need hardly stop here to explain the position of this journal in regard to genuine American improvement in medical science, especially in practical surgery. It is not our intention to underrate the value of *metallic* sutures, or to dispute the merit, which belongs to Dr. Sims, of having introduced their more general employment in the management of wounds and plastic operations. Still less do we wish to detract from the honor due to him for the material advance in methodizing and improving the details of the particular operation to which he owes his professional success. It will not be difficult to show that, in these matters, Dr. Sims himself, in his anniversary oration, has proved his worst enemy, and the first one to attack the laurels which have hitherto been generally awarded to him, and by none more fairly and frankly than Dr. Bozeman. He does this so gratuitously, and in a temper so evidently the effect of jealousy, that we heartily lament his misfortune in having had no thoughtful friend to suggest to him, or his academy admirers, the maxim addressed to the cobbler of old.

*Ne sutor ultra crepidam* was never more applicable than to the course of the self-gratulating laureate of the "Silver Suture." His achievements, with the vaunted stitch and its accompaniments, were willingly accepted as a material accession to the triumphs of surgery, in behalf of suffering womankind under one of her heaviest and most disheartening afflictions; and the champion of the suture was hailed as a master, and requited in fair proportion to the good he had effected by his perseverance, ingenuity, and skill.

Not satisfied, however, with the brilliant reputation obtained through the legitimate account of his investigations and their then unparalleled results, he was tempted, by a dread of divided honors, to stoop to trumpet his "discoveries" and rail at his "followers," before a mixed audience, which must have been much more amused and disgusted than it could have been instructed.

In the midst of his success he had found a Mordecai at the palace-gate, whose humble but decided merit in the same field offended him. He waxed eloquent, not only in sounding his own praises "from the housetops," as he says, but in denouncing the troublesome competitor who had dared to improve upon his instruments and mode of operating. He strove to suspend this too fortunate rival on a gallows of professional disgrace by accusing him, without proof, and in direct contradiction to the published evidence, of ingratitude and plagiarism. In the effort to disparage the single but important step in advance, established by Dr. Bozeman, he perpetrates the folly of stripping his own operation of its characteristic features, by destroying the admirable combination of other men's methods, in which the superior advantage of his former plan consists; while he commits the very crime he would fasten on his quondam associate, by ignoring the inventions of his predecessors and clamoring for the sole credit of what he certainly did not originate.

We purpose to show that he acts very differently from Dr. Bozeman in his treatment of Mettauer and Levert, of this country, and of Dieffenbach, Velpeau, and others, abroad, by the magniloquent exaltation of his own genius at the expense of their rights; and that he even deigns to appropriate a hint from the arch offender, Dr. Bozeman, himself.

Let us not be misunderstood as to our view of the right of property in scientific inventions and their application. We acknowledge little interest in the vain and petty quarrels for precedence in the honor of inventing new instruments and modes of treatment, or even in the discovery of scientific truths. After all, he is the greatest benefactor who succeeds in teaching his fellow-men the actual use and value of a new step in art or science, whether or not his happens to be the first glimmer published or put on record, much less imagined, of its existence or availability. This is virtually the world's verdict, in spite of the often-repeated reclamations for theoretical priority which are so vehemently asserted at the eleventh hour; and we believe it is the just one. But the true leader of humanity, whose intelligence and energy have enabled him, by outstripping others, to win the prize, can well afford to acknowledge the advance already proposed by his less active predecessors, and the aid he may have derived from their suggestions; nor has he anything to gain from an attempt to stultify his own advance, and bar further progress, by resisting improvements on himself, although those improvements may throw his "into the background."

"SILVER SUTURES IN SURGERY," says Dr. Sims, "is a subject that necessarily involves frequent and constant reference to my own labors and their results; indeed, it has been the theme of my life for the last twelve years. In selecting it, I am not ignorant of the delicacy of my position, and of my liability to be misinterpreted, and to be criticised by some who do not know me as you do. Nevertheless, I shall speak as an American—plainly, frankly, and fearlessly, feeling that you and the great mass of the profession will understand and fully sustain me. So far as it concerns my experience, personal narrative, claims as a discoverer, or defence against aggression, I have a right to declare them openly 'from the house-tops;' and for this, in the abstract, I hold myself responsible. But whether the subject-matter will be considered quite appropriate for the occasion, is a question only for the fastidiously censorious; and I shall dismiss that very summarily, by shifting its responsibility to the broad shoulders of the gentlemen just named, at whose solicitation I stand here to-night."—pp. 7, 8.

We should be very loathe to believe that the distinguished members of the Academy of Medicine, here alluded to, would knowingly subject themselves to such a burden as Dr. Sims has cast upon them, in return for their good-natured confidence in his sense of propriety as well as professional experience. We shall not question their opinion as to the fitness of the "subject-matter" for an audience containing many men and women incapable of understanding anything about it, except the revolting and once hopeless nature of an infirmity to which one sex is liable in its most important functions, and the vast debt of gratitude which both sexes owe to the individual before them for the "only genuine cure." But we do not believe that these officers of the Academy can contentedly accept the compliment which thus compels them to indorse the self-glorifying appeal, over their shoulders, to a miscellaneous assembly. It is far too likely to remind them of the large-lettered exhibitions which are suspended from the backs of a much humbler class of peripatetics in their public streets.

Not having the fear of the speaker or his sponsors before our eyes, we do not hesitate to range ourselves among the "fastidiously censorious;" and we venture to express the hope that the hitherto interesting series of annual mementoes, which began so well with Dr. Francis and had reached a climax not easily surpassed in the scholarly address of Dr. Watson, may cease altogether, rather than that such perversions as this of Dr. Sims should be allowed to threaten the well-earned fame of the Academy.

"You who are familiar," continues our author, "with the experience of that noble charity, the Woman's Hospital, will not be surprised, when I declare it as my honest and heartfelt conviction, that the use of SILVER AS A SUTURE IS THE GREAT SURGICAL ACHIEVEMENT OF THE NINETEENTH CENTURY.

"For my country I claim the honor of this imperishable discovery, and seize this auspicious occasion to place permanently upon record a history of its origin and progress.

"Many of you already know that it was not the result of mere accident, but of long, laborious, and persevering effort, based upon the immutable principles of science, and forming one of the most beautiful examples of inductive philosophy.

"Wishing to impress upon the profession its importance and value in general surgery, as well as in injuries from protracted parturition, I shall necessarily be compelled to draw largely and somewhat minutely upon my past experience. But this will be readily tolerated by a liberal profession; for, while I labor to establish principles, it will be legitimate to refer to dates, and times, and places, and persons, and circumstances, all of which are necessary, to place forever beyond cavil my claims and agency in this discovery.

"In 1845 I conceived the idea of curing vesico-vaginal fistula, and entered upon the broad field of experiment with all the ardor and enthusiasm of a devotee. After nearly four years of fruitless labor, silver wire was fortunately substituted for silk as a suture, and lo! a new era dawns upon surgery.

"This was on the 21st of June, 1849, since which time I have used no other suture in any department of surgery."—pp. 8, 9.

In another part of the pamphlet, after assuring us that every case of vesico-vaginal fistula "is easily and perfectly curable that has tissue enough to render any operation whatever practicable, while a failure is the exception to the rule," and that there is in these operations "not the least risk to life," he raves on in the following strain:—

"I am not claiming too much for this suture when I say that the same relative result must be attained in all other surgical operations requiring sutures, if the same method be adopted.

"My language is in nowise extravagant; and I shall yet live to see the day when the whole profession of the civilized world will accord to this simple discovery the high position of being the most important contribution as yet made to the surgery of the present century.

"The only thing at all comparable to it is Etherization; and in practical results of permanent benefit it is absolutely contemptible, when compared with those from the universal use of silver sutures in the broad domain of general surgery."—p. 46.

In the foregoing extracts Dr. Sims has the benefit of a sufficiently full statement, *verbatim et literatim*, of his position on the suture question. Let us see what is his idea of the right of ownership in the grand invention.

"As concentrated efforts have been made in various quarters to rob me of full credit for my labors, I have thought it due to truth, to justice, to posterity, and to myself, to place permanently upon record a history of the circumstances attending this discovery."—p. 46.

These various moral, personal, and prospective interests are then duly sustained in a long and extremely characteristic "personal narrative," which cannot fail to divert and astonish the reader as much as it interests him. For the present we pass over the details of the early efforts, the gradual but laborious unfolding of new plans, the numerous discouragements but steady progress toward improvement, and the crowning triumph to the years of toil, which do him so much honor. Some excitement may be well excused in one who chronicles the checkered fortunes of a struggle fraught with so much that is laudable in its conception and career. But the vain-boasting and unfairness of our hero provoke a sifting of his story, and lead us to seek human agency for the sources of inspiration which he describes as "Providential." This agency may be fairly attributed to Wurtzer, Chelius, Velpeau, Dieffenbach, and Churchill, and to Hayward, Mettauer, and Pancoast, whose different suggestions and expedients afforded the needful hints to choose from, without the necessity for higher revelation. The real heroism is to be found in the all-trusting women who went on with never-failing fortitude, enduring trial after trial, to enable this one man to work out upon their lacerated bodies an almost hopeless problem which was to determine the life-long happiness or misery of multitudes of their sex. Theirs was the great act; and, much as they have gained in the escape from suffering, let them still share the praise. This share is graciously allowed them by Dr. Sims in the following strain:—

"To the indomitable courage of these long-suffering women, more than to any one other single circumstance, is the world indebted for the results of these persevering efforts. Had they faltered, then would woman have continued to suffer from the dreadful injuries produced by protracted parturition, and then should the broad domain of surgery not have known one of the most useful improvements that shall forever hereafter grace its annals."—p. 55.

To return to our "narrative;" after years of fruitless experiment he finally imagined that the only remaining obstacle was in the irritating action of the material of the suture.

"Now, the question arose, was there a substitute for silk that would answer the same purpose, and yet not poison the animal tissue? Why, lead remains indefinitely in the body, becomes sacculated, and produces no poisonous or suppurative effect; Dr. Levert, of Mobile, (*Am. Jour. Med. Sci.*, No. VII., May, 1829,) had demonstrated the innocuousness and efficiency of leaden ligatures on the arteries in the lower animals, and Mettauer and Dieffenbach had actually used leaden sutures in these very cases; and I had in my various experiments tried them in two cases of vesical, and one of rectal fistula, but, fortunately for science, the clumsy leaden wire was unsuccessful in my hands. Was there any other metal that could be substituted for lead, possessing its valuable property of harmlessness?

"In the train of inquiry what would be more readily suggested to the reasoning mind than silver, gold, and platinum?"—pp. 59, 60.

He tells us he has used all of them, but adopts the silver, "because it is as good as gold, and cheaper." Just at this crisis he happens "to pick up a piece of brass wire that had been used in a pair of old-fashioned suspenders made before the days of India rubber; it was fine as ordinary sewing thread." He got a jeweller to imitate this in silver; and, on the 21st of June, it was applied, with leaden bars and the perforated shot, to a young colored woman, who "had never murmured at the preceding failures," and was "placed on the operating table for the thirtieth time."

"In all previous operations the urethra, in a day or two, would become red and tender, and the urine loaded with thick tenacious mucus, thus showing the inflammatory process, which was adverse to union; but after this operation, the urine remained perfectly limpid all the time, and on the eighth day the parts were perfectly healed; the suture apparatus remaining just as it was placed, with the cross-bars somewhat burrowed in the vaginal tissue." At last he had attained what he had "worked for for nearly four years; and it was but a few weeks before all the cases were cured that had been the subject of experiment for so long time."—pp. 60, 61.

This termination of his gropings was happy enough, one might suppose, to admit of some little gratitude to the few investigators whose previous inklings and recorded experience were entitled to at least as much notice as the old suspender wire. He mentions Levert, as having experimented on arteries with *leaden* ligatures, but neglects to tell us that Levert experimented with *silver, gold, and platinum wires, as well as lead.* He might have remembered, too, that silver wire was long ago advised and used by a member of the New York Academy, (Dr. J. Kearney Rodgers,) as a suture between fragments of bone, and has ever since been familiar to American surgeons. Whether or not it is new as a suture for soft parts, its employment as a means of retaining fragments of bone in apposition dates at least as far back as the eighteenth century.

This question, however, is of trivial importance; we should not have mooted it with one less grasping in his demands, and more liberal to his neighbors in minor matters, than the author of this address. Dr. Sims deserves and receives the usual credit for developing the advantages of the metallic suture, and for urging its application in many cases in which he was, doubtless, the first to resort to it and demonstrate its value. An ingenious modifier and manipulator, untiring in the pursuit of the required expedient, and able to appreciate its application, he certainly is; but, with all this inventive capacity, he has yet to establish a foothold in the field of independent scientific "discovery."

Admitting, then, his position as the author of silver suture surgery, we still want to know what mysterious virtue there is in virgin silver to make it the *sine qua non* in keeping wounds in proper order. How has it been shown that cheaper, more pliable and stronger metals are not equally available, or cannot easily be made so by coating with silver or with other unirritating material? Galvanic action might interfere, but who has shown this? If it be true that metallic ligatures or sutures are in all cases preferable to animal or vegetable—which we beg leave to doubt exceedingly—then let iron, the cheapest, strongest and most pliable when finely drawn, be fairly tested, with and without a covering. We have tried electro-plated iron wire and found it more delicate and convenient than the silver wire which had been provided for the case. We doubt the necessity, however, of plating the iron wire at all; it might be tinned, but would probably do just as well uncovered, since the common twisted suture needle has been long enough in use, in hundreds of delicate plastic operations, to prove its unirritating qualities and freedom from injurious oxidation. Copper, (the best for plating,) tin, and aluminum, also demand a trial, as well as iron and lead.

Dr. Sims refers to lead as having been employed by Mettauer and Dieffenbach; but, although unwilling to say anything of the labors of these operators, he quotes his own failures with their suture only to disparage it, and dismisses it as "clumsy." These negative results and the cavalier dismissal can hardly counteract the authority of Mettauer and Dieffenbach. (See Mettauer's cases in *American Journ. Med. Sci.*, January, 1833, and April, 1837; also Dieffenbach in same Journal, Nov. 1838.) Nor does the idle epithet indulged in affect the applicability of the vulgar metal, *when properly prepared and handled,* to all the legitimate functions of the more stylish substitute of Dr. Sims; still less does it deprive the leaden thread in the hands of Mettauer and

Dieffenbach, of the post of honor, such as it is, in the van of the metallic sutures.

We have neither time nor space to follow Dr. Sims in his harangue upon the vastly superior action of his silver suture in various surgical cases, or to examine into the need of any kind of suture in many of the dressings for which he recommends it; nor do we wish to reopen the very old debate upon the good and bad effects of silken sutures, which has been going on, with frequent fluctuations but no material practical conclusion, ever since the time of Pibrac and Louis, of John Hunter and John Bell. The new turn and impulse to the discussion, given by the fancy of Dr. Sims, is of less importance to the management of external wounds in these days of collodion and isinglass adhesive strips; and yet suggests a good deal of experimental inquiry which ought to be pursued, and which would have added greatly to the value of his paper.

So much for the first object of the anniversary address. The second seems to be to demolish Dr. Bozeman and his "button suture," which latter had become a stumbling-block to Dr. Sims.

This branch of the argument begins, directly after the grand announcement of the dawn of the "new era,"—the silver age of surgery,—with a reference to the speaker's article, in the *American Journal of the Medical Sciences*, on "The Treatment of Vesico-Vaginal Fistula," as containing the first revelation, in the shape of "full and specific directions for every stage of the operation." "The silver suture as then used," he says, "I called the 'clamp-suture,' on account of its method of action in forcing or 'clamping' firmly together the surfaces to be united." He then describes the component parts and the action of this quondam clamp arrangement, and gives a wood-cut in illustration, "merely for its historical value, and to show the progress of improvement."

Everybody understands that the new surname of *clamp*, thus given to an old form of suture, was very generally received, because it does convey a better idea of the well-known action of the quills, and because it represents a neat improvement of the primitive cylinders, which the author of the name had unquestionably contrived. We are sorry to see the Doctor thus casting off what, in spite of his own protest, will continue to be regarded as an inseparable feature of his particular method. Whether superseded or not by other appliances, in addition to the metallic suture, it cannot be replaced, either in name or function, by the suture in itself as the only essential part. The clamp, although applicable only to fistulæ of Velpeau's second and third classes, and utterly unfit for his first and for the fourth and fifth of Bozeman, has done too much good service in other hands, as well as those of Dr. Sims, to be altogether set aside at this stage of its history. Yet Dr. Sims assures us (p. 21) that "the great success of these operations" [including Bozeman's,] "is due entirely to the silver wire. I had long ago demonstrated, over and over again, that the clamps or leaden bars and perforated shot were totally worthless, if used with silk as a suture; in other words, that the silver wire was the essential part, the *sine qua non* of success." After adverting to some disparaging experience with Dr. Bozeman's suture, in which he appears to have been strangely awkward and unfortunate, he informs us that "seeing thus that the much vaunted button was obnoxious in some cases, and nugatory in others, I now began a series of experiments with the wire as an interrupted suture, without 'clamps' or 'buttons,' so-called." He had found it excellent in general surgery; and he saw no good reason why it should not answer equally well in the management of the fistulæ on which he was then engaged. It was accordingly applied on the 24th of June, 1856.

This was about six weeks after the intruding "button" was presented to the profession, and betrays an awakening sense of weakness in the clamp, which, until the entrance of the new light, its cautious advocate had not ventured to indulge. Similar experiments, however, had been long ago performed with promising success by Dieffenbach and Mettauer, the only difference being that they used lead, which we believe to be better than silver as an interrupted suture.

Curiously enough, and very unhappily for his own way of thinking from the *suum cuique* stand-point, the first steps of his revised plan of operation are precisely those of Dr. Bozeman; while the twisting of the wires which completes the union, as also the method of withdrawing them, are the processes of Mettauer; except that the wires are cut off by Dr. Sims, whereas Mettauer, although he cuts them off in staphylorraphy, more wisely leaves them long enough to reach the entrance of the canal. The wires are drawn together by an instrument shaped and cleft like the broad end of a French director, which is evidently a modification of Bozeman's adjuster. In short, the new "operation" is another very pretty illustration of the adaptive power of our enterprising but too captious orator. We acknowledge that these are very little matters of dispute;—as unbecoming to the pages of a respectable review as they are to those of a scientific essay or annual address. Our object, in dwelling on such questions, is simply to suggest to Dr. Sims, in future discussion of other men's labors in a field which is still open to all, an application of the golden rule.

To return to the story of our amiable and modest author:—

"In my own hands," he continues, "this method of using the silver wire" [*i.e.* with clamps and shot,] "in vesico-vaginal fistula, was uniformly successful, because I always took good care to make a broad and free scarification of the edges of the fistula, and to pass the sutures so far from them that the cross-bars or clamps would burrow in the vaginal tissue, there to remain till the case was permanently cured. But my followers were not so successful, simply because these two important points were not fully appreciated. They complained that the sutures would cut out; a thing that never happened with me in but three or four cases, and they were among my first experiments, before learning thoroughly the art of applying them."

Dr. Sims here forgets his own experience, especially as recorded in his former article, (*Am. Jour. Med. Sci.*, Jan., 1852, p. 70,) in which, in addition to the cutting out and other "difficulties" requiring "great judgment, which experience alone can give, to counteract," he speaks of having witnessed "in two or three instances, a still more serious accident from an undue pressure of the clamps, viz. a strangulation of the inclosed fistulous edges, which unfortunately resulted in a sloughing of the tumefied parts, and a consequent enlarging of the opening."

The charge against his "followers," of ignorance, is an unsustained assertion, in relation to which, Bozeman and the now comparatively numerous operators are entitled to an equal footing with himself. It is well contradicted by one of his ablest and most experienced commentators (Dr. Kolloch) in the following remarks:—

"The wires *will* cut themselves out in certain cases, however much attention may be bestowed on their introduction at a sufficient distance from the edge of the raw surface, and sufficient depth into the submucous tissue; the lips included between the clamps will slough, however much judgment may be exercised in drawing them together, and irregularities on the vaginal surface, rigidity from cicatrices, and the situation of either a part or the whole of the fistulous opening, may prevent the clamps from being evenly applied, and with sufficient parallelism to secure their regular and efficient action. In consequence of these occurrences the patient has to be subjected to a greater or less number of repetitions of the operation; and, perhaps, other means have to be employed for the perfection of the cure." (Report on the History and Treatment of Vesico-Vaginal Fistula, read before the Georgia State Med. Soc., p. 17; published in the *Savannah Jour. of Med.*, and in the *Southern Med. and Surg. Jour.*, Augusta, Georgia; see also a Review by H. R. S., in *Am. Jour. Med. Sci.*, Oct., 1857, p. 392.)

The same authority, in a recent review of this pamphlet of Dr. Sims, adds some further testimony to his previous evidence in favor of the superiority of Bozeman's plan.

"The attempt of Dr. Sims to disparage 'the button' will not readily succeed in producing conviction in the minds of those who have had an opportunity of comparing the two methods. We have not the pleasure of the personal acquaintance of either Dr. S. or Dr. B., and have, therefore, no personal predilection to bias our judgment.

"Before we were acquainted with Dr. Bozeman's Modification of the Silver Wire Suture, we had operated several times unsuccessfully with Dr. Sims's clamps; but as we knew of no more perfect method, as we were blessed with a pretty good stock of patience and perseverance, as we found that the fistulæ (although not cured) were very much contracted by each operation; above all, as we had, like Dr. Sims, willing patients at our control, we were determined to go on, yea, even to the thirtieth or fortieth repetition. Before, however, our long-suffering patients were subjected to this severe ordeal, we were furnished by Dr. Bozeman with his pamphlet, and lo! success crowned our very first trial.

"With such evidence as this before our eyes, could we refuse to acknowledge the superiority of the button over the clamps?" (*Savannah Jour. of Med.*, May, 1858, p. 59.)

Dr. Mettauer, who has "treated and cured *twenty-seven* cases with the leaden interrupted suture alone, and without the least difficulty," in an instructive paper, (published June, 1855, before the appearance of Dr. Bozeman's article,) after stating that the "quilled suture, as improved by Dr. Sims, had not succeeded in his hands," holds the following language:—

"In each case, three in number, the operation failed from ulceration of the denuded borders, both at the margins and where the metallic clamp rested on them, and by the premature cutting out of the silver wire.

"In one case the clamp induced sufficient ulceration to form a new opening into the bladder so early as the fifth day after the operation. Possibly I may have used undue force in the approximation of the denuded borders in these cases; but it seemed to me that I only carried it to the extent of placing them in complete and firm contact.

"This suture, however useful it may have been in the hands of Dr. Sims, is liable to important objections. If applied with two much force it will almost certainly cause ulceration or actual sloughing of the denuded borders; and it will be very difficult, in most cases in which it is used, to determine the degree of force which should be used in applying it." (*Virginia Med. and Surg. Jour.*, June, 1855, pp. 451, 452.)

One more testimonial may be offered here, (from the review of Dr. H. R. Storer, in the *Am. Jour. Med. Sci.*, loc. citat.,) which we quote in full because it presents an excellent and appreciative summary of Dr. Bozeman's labors, and because it is free from the suspicion of undue influences to which our own remarks may be subjected.

"It is to Dr. Bozeman, of Alabama, to whom it accidentally suggested itself, that we are indebted for the long-looked-for discovery, now known as the button suture. His first paper was published in the spring of 1856, and he has lately made known the results of a more extended experience, by diagrams, accurate descriptions, an elaborate classification of all possible varieties of fistula, and directions for perfectly adapting his apparatus to each and every one of them.

"The early stages of his operation are identical with those already described.

Silver ligatures are used, but are introduced directly and without the aid of any other thread. Instead of being fastened to metal quills, the extremities of the wires, brought together like Mettauer's before twisting, are passed through minute perforations in a shield of lead, which is found to answer much better than the silver at first proposed, and, as by Sims, clamped securely with shot.

"It is claimed that the metal shield will—better than Sims's cross-bars—1. Act the part of a splint in keeping the approximated edges in close contact, and at rest; 2. Prevent the wires from cutting out; and 3. Protect the edges of the wound against irritation by the urine, vaginal discharges, or atmosphere.

"The button suture has been fairly tried. Successful cases have been reported, besides the fifteen [now twenty-four] or more of Bozeman, in this country, by Gaston, T. Wood, Kolloch, Williams, and others; and in Great Britain, by Spencer Wells and Baker Brown; all of whom corroborate its excellence. Kolloch's report, indeed, prepared evidently with care, and well and candidly written, is mainly occupied by cases from his own practice, show the relative merits of the clamp and button. Dr. Bozeman has found that the more difficult cases of ordinary fistula can be easily cured, but there are two lesions he has mastered which have hitherto been entirely beyond surgical aid.

"The first of these is a longitudinal laceration of the edge of the meatus, [termed '*rent of the urethra*,' by Dr. B.] 'the most unfavorable form of all the urethral injuries,' for which no treatment had ever been proposed.

"The other victory alluded to is in those cases where the cervix uteri is directly involved in the fistula. Nothing had been done unless by Vidal's method of closing the vagina, save by Jobert, who, until lately, by extensively dissecting away the attachments of the cervix, whether or not accompanying this by the insertion of a flap from a distant part, managed to close the fistula but lost his patients by peritonitis.

"Bozeman, on the other hand, claims better fortune. By his method the uterus itself is dragged down, the edges of the cervix are passed, just as with any part of the vesico-vaginal septum, and stiches inserted into its substance.

"The idea of this bold procedure, as novel as it is successful, had undoubtedly presented itself to the mind of Velpeau, (*Operative Surgery*, vol. i., p. 627; see also his *Clinique Chirurgicale*, vol. ii., p. 267,) who speaks of the possibility of dragging down the cervix and making it subservient to closing the fistula, but remarks, 'all these suggestions want a foundation to rest upon; none of them can yet adduce any success in their favor;' and Jobert, improving upon himself, relates, at the close of the year, a case in which sutures were passed through the cervix; (*Union Médicale*, Nov., 1856;) but the credit of having independently conceived the operation in all its completeness, and of having put it into actual practice, is undoubtedly Bozeman's."

This review also mentions a unique and invaluable application of the button, which deserves especial notice. Speaking in praise of Sims's catheter, as indispensable, he says:—

"In those lacerations of the meatus already referred to, this instrument cannot be borne; but by an ingenious arrangement of his button-shield, Bozeman has compelled it, though designed for an entirely different purpose, successfully to take the place by affording the necessary support to a male elastic."

The reader is now prepared to appreciate the wisdom and delicacy as well as justness of the attack of Dr. Sims upon the unwitting offender who had left him so far behind in the study of their common theme.

In 1853, being obliged by ill health to leave Montgomery, Alabama, Dr. S. "gave Dr. Bozeman of that place a partnership in business,* and indoctrinated him in my peculiar method of operating for vesico-vaginal fistula, instructing him in my various modes of using silver wire as a suture, not only in this class of affections, but in general surgery. Not understanding its principle of action, and, therefore, failing in its practical application, he was quite disheartened with his ill success, when by mere accident he fell upon a plan of fastening the wire, and so modifying my method, that in awkward or inexperienced hands it became easier of application. Instead of passing the wires through the leaden bars on each side of the fistula, he passed them through a concave disc or 'button' which rests upon the surface of the parts to be united."

In the marvellous history of his own discoveries he stumbles on the best mode of looking into the vagina, by a "Providential incident;" and announces (p. 51) that "THERE ARE NO ACCIDENTS IN THE PROVIDENCE OF GOD." It seems, however, that the device of Dr. Bozeman is not to be thus canonized, although it had neither precedent nor incident to bring it into active being. By mere accident only did he fall upon a plan, which could not be respectable because it so modified his master's method as to make this "easier of application," and thus bring it within the unhallowed reach of "awkward and inexperienced hands!"

The balloon experiment of the kneeling patient, so graphically recorded, (p. 50,) in which the whole vaginal expanse displayed itself for the first time to the admiring eyes of Dr. Sims, was one which, although not thus explained, was well known to surgeons of any reading, long before it thus occurred to the practical mind of our historian. The position on the elbows and knees suggested by it was years ago recommended by Wurtzer, Chelius, Velpeau, and Churchill, and is quoted by Pancoast in his *Operative Surgery*, published in 1844. Like the unlucky clamp, however, this other once prominent feature in the process of the Doctor is withdrawn from its place of honor by the immortal stitch. A position, partly prone and partly on the side, much less constrained for the patient, and, as we believe, from one trial of it, sufficiently convenient for the ope-

* [The partnership referred to, we understand from Dr. B., was only nominal, and was formed, at the earnest solicitation of Dr. Sims, for a special purpose and consideration, just as he was making his arrangements to come to the North, and not longer than two months before his departure.—EDS.]

rator, is now recommended. It is illustrated with a full-length picture which is not equal to many of the others, either in design or execution.

Our orator claims to have "had a mission, if not of a Divine character, at least but little short of it,—of Divine origin;" and to be engaged in "a labor of love under Divine guidance for the furtherance of a truly benevolent purpose." In a season of discouragement, too, something told him "that the work had to be done, and that if I should fall, God in his wisdom would raise up some one as an instrument to carry it forward to a glorious consummation."—pp. 54, 55.

Better times have not increased his Christian humility or charity. He evidently wishes to remain alone in his solemn glory, and will brook no son of Jesse near the throne.

"Notwithstanding the fact that the Doctor lived in Montgomery for years, without any professional position till I gave it to him, that he is indebted to me for what he could never have obtained without my aid,* he appropriates to himself every step of the operation that resulted from my own individual and unaided efforts,—even my silver wire and perforated shot, the only things of any real value whatever, and publishes it as his operation by a 'new mode of suture,' making strenuous efforts to place my labors in the background.

"I do not complain of modifications, but I do complain of a disingenuousness that would be dishonorable even under widely different circumstances."

The pathetic earnestness with which, like the Israelite of Venice, he thus bewails his daughter and his ducats, would only rouse a smile, if the outcry were not burdened with a serious personal charge.

In order to contradict this charge at once, we have only to cite a passage from Dr. Bozeman's first paper. In objecting to the clamp suture he expressly states that,—

"I do not wish to be understood as attempting to detract from the great credit due, from the profession and the public, to Dr. Sims for his untiring perseverance in bringing his method to its present high state of perfection; I consider that this gentleman is fully entitled to all the praise that has been bestowed upon him, both in America and Europe. To the honor of his professional brethren in this country, it may be stated that no one has been found who has not gladly accorded to him the high distinction that he at present occupies. I am sorry that the same cannot be said of European surgeons in general; for, with the exception of Mr. Erichsen, Mr. Brown and Mr. Druitt, of London, no one on the other side of the Atlantic has proved sufficiently frank to do full justice to Dr. Sims's claims. Fully impressed, therefore, with the importance of the position I assume in attempting to show that the clamp suture is objec-

* [Dr. Bozeman had the prospects of any other young practitioner of his age and acquirements. The only professional position granted, that we have heard of, was the dwelling-house and office of Dr. Sims, for which the latter received what was considered, at the time, an extravagantly high price.—EDS.]

tionable, I proceed to the task, actuated, as every inquirer after truth should be, by no other motives than a desire to make facts and principles subservient to the great ends of science."

Dr. Bozeman does not claim originality, as he might well have done, for his button, but offers it only as a modification of the twisted suture. In our view it is altogether unlike and superior to the twisted suture, being capable of greater variation and serving more completely as splint, shield and director, all in one. The only thing that approaches it is the *brad suture;* but that is only a convenient species of quill or clamp suture, with few of the mechanical advantages of the various forms of button. As for the outcry about the non-acknowledgment of "even the silver and the shot," it is sheer nonsense to suppose that Dr. Bozeman was bound to say, in so many definite words, what had long been a matter of notoriety among professional men. His general tribute to Dr. Sims's pre-emption rights was ample, and, of course, included all matters not expressly mentioned. But Dr. Bozeman can easily do without the shot as well as the silver; for he only needs a furrowed ridge upon the outer face of the shield along the line through which the holes are to pass, to secure a fixed and ready means of clinching the wire, which is better than the "clumsy" and slippery contrivance of Dr. Sims.

Dr. Sims, in narrating a hare-lip case, describes an arrangement of softened ivory, or a softened and flattened piece of quill, to be applied as a shield and a support under the wires, which is clearly suggested by Bozeman's disc; which indeed had actually been proposed as such, in private practice, by two different surgeons of our acquaintance. Although engaged at the time in exalting the silver suture at the expense of the shield, he thus impliedly admits the advantage of the latter, but utters not a word as to the source of his suggestion.

This very attempt, perhaps unconscious, on the part of Dr. Sims, to avail himself of the principle of mechanical support and retention to which the broad disc is so well adapted, shows the necessity, which he perceives, for something more than the barren and negative quality of non-irritation, which alone distinguishes the metallic suture from other sutures, as a bond of union. Beyond the absence of superficial irritation, its ordinary use involves no "principle" whatever that is not common to silk and flax, and that is not surpassed in rational attributes by other modes of retentive dressing.

On the other hand, the button shield, as already shown in part by Dr. Storer, is really philosophical, and is capable of application to an abundant variety of solutions of continuity. This very capability would lead an intelligent and earnest practitioner to classify the forms of injury to which it may be adapted. That this has been done in fistulæ with great care and clearness by Dr. Bozeman, is much to his honor, since he has thus effected more scientific progress than with any other portion of his labors.

In the course of his investigations he has ascertained the relative frequency of certain fistulæ, and has learned that the most prevalent (about thirty per cent.) are those involving the neck of the uterus, his fifth class, and one in which the clamp is altogether unavailable.

It was not our expectation, however, to consider Dr. Bozeman's plans and principles of treatment. They are fully given in his several papers, especially the last, already published in this journal. Our sole desire has been to expose the folly and injustice of the attack upon his honor as a man and a physician, by the one who should have cherished him the most, not as a rival but as a worthy coadjutor. Nor need we follow the aggressor in his feeble and inconsistent strictures on the "principle" of the button suture, and in his misconstruction of its application; still less in his ill-judged and inconclusive trials of it, as exhibited in his discourse.

Of the six cases subjected to the pretended test and found wanting, three were cases of operation for occlusion of the vagina, in which the button is not recommended, and in which a different operation should have been performed; while not one of the operations in the other three was performed in accordance with the precepts of the inventor.

Before parting with Dr. Sims we would humbly ask his leave, and, if need be, that of Drs. Bozeman and Mettauer, to propose another "modification." From what we have seen of leaden ligatures, and of these vesico-vaginal operations, we are strongly prepossessed in favor of Mettauer's suture. Dr. M. thus describes his method of using this, after having introduced the several threads, directly opposite each other, through all the coats of the bladder and vagina, "fully an inch from the denuded margins on the vaginal surface":—

"I next proceeded to approximate the divided borders, by making traction on the metallic threads, and then carefully confining them in close and exact contact, by twisting their free ends together, until the loops were sufficiently abridged to enable them both to support and moderately to compress the structures embraced by them; and for the purpose of twisting the wires I employed a light pair of self-closing forceps, without ring-handles, having found that instrument far more handy than the ordinary dressing forceps, or forceps with ring-handles. After securing the first suture so as to bring the denuded borders well together, I tightened the second, and was gratified in finding that the fistulous opening was perfectly closed, and that not a drop of urine escaped between the borders. I was careful, in twisting the wires, not to employ undue force, but sufficient to bring the opposing surfaces into complete and firm contact, without injuriously compressing the unlooped structure; and I invariably twisted from left to right, to prevent confusion.

"After many trials in determining the extent to which the tension should be carried, I finally adopted, as a safe rule, the fixed and erected state of the twisted extremities of the wires, and their bristle-like spring when touched with the probe; and the tightening of the loops should be executed with extreme caution and gradually, testing it, as we proceed, from time to time, by touching the wires with a probe or the extremity of the forceps."—(*Virginia Med. and Surg. Journal*, June, 1855, p. 449.)

According to his experience, it is necessary to tighten the ligatures on the second or third day after the operation; which can be most readily done by simply twisting the wire a few turns, until the approximating force is once more steadily exerted, as shown by the spring-like tension of the twisted wires above described. The ability thus to graduate the approximating force, and subsequently to renew this approximation, if necessary, constitutes, in Dr. Mettauer's view, a very weighty source, among others, of superiority in the interrupted leaden suture over the clamp. The leaden suture has this advantage, to some extent, over the simple interrupted silver suture also, because of its greater pliability; but the twisting manœuvre is very easily adapted to the shield of Bozeman, and renders this latter especially desirable for the purpose. All that we need is a double row of holes parallel to each other, instead of a single one as heretofore. Passing the two ends of each ligature through their corresponding pair of holes, we would then twist their ends together gradually and cautiously, as directed by Mettauer, and we would repeat this twisting, or release the wires by untwisting, according to circumstances in each case.

In order the better to watch the condition and relative positions of the sutured parts, we would prefer a transparent horn or tortoise-shell shield to the lead at present used. This transparent shield, with parallel lines of perforations, and a series of slender leaden or iron or tin wires properly applied, and to be twisted, retwisted or untwisted, *pro re nata*, might be made to answer every indication in hare-lip and other plastic operations; at least, as well as the flattened quill and silver suture plan proposed by Dr. Sims. We again beg permission to submit our *horn-button and lead, iron or tin suture*, for his especial and candid consideration.

The contrast between the combination of vile things in our "invention" and the *simplex mundiciis* of their lordly rival is, doubtless, painful to eyes and ears polite; but, until we are convinced that silver is entitled to monopolize the position as a connecting medium in wounds which it holds as a circulating one in trade, we must continue to adhere to the more serviceable iron and lead, or, peradventure, TIN.

In conclusion, we again regret that the New York Academy of Medicine has permitted this publication to go forth under the sanction of its seal and the formal action of a committee composed of some of its best men. It was bad enough, in all conscience, to oblige these gentlemen and their fellows to listen even to its delivery, not omitting the exordium to the "presidential galaxy" with which the orator apologizes for the intrusion of his private griefs. But to involve any public body, through an annual address, in the endorsement of such a tissue of adulation and self-glorification, is an imposition on its character for taste, judgment, dignity, and ethics, which ought not to go unwhipped of justice,—least of all by a society which ranks among its members many of the brightest ornaments of the American profession.

In regard to the author himself, we are satisfied that, whatever he may think of our own remarks—which have been indited in no recriminating spirit, as we have no personal interest in either party—he must deeply lament the occasion which has thus brought him so unpleasantly before his brethren and the public. Our task has been a thankless one, as unpalatable to ourselves as it may be to him; and we heartily wish that he and his reviewers could have been spared the necessity of its infliction. To our readers we have to say, in excuse for troubling them, that in the performance of the censor's duty we have striven to respect the adage,—"If offence come out of truth, it were better that the offence come than that the truth be concealed."

